# Design and Evaluation of PLGA-Based Hybrid Nanoparticles for Nasal Paclitaxel Delivery: Formulation Optimization, Barrier Transport, and Cytotoxicity Assessment

**DOI:** 10.1021/acsomega.6c05503

**Published:** 2026-08-30

**Authors:** Anna Carolina da Silva Sousa, Caroline Mari Ramos Oda, André Luis Branco de Barros, Maria Betânia de Freitas Marques, Chun Yuen Jerry Wong, Hui Xin Ong, Daniela Traini, Elaine Amaral Leite

**Affiliations:** † Department of Pharmaceutical Products, Faculty of Pharmacy, Federal University of Minas Gerais, Av. Antônio Carlos, 6627, Belo Horizonte, Minas Gerais 31270-901, Brazil; ‡ Department of Clinical and Toxicological Analyses, Faculty of Pharmacy, Federal University of Minas Gerais, Av. Antônio Carlos, 6627, Belo Horizonte, Minas Gerais 31270-901, Brazil; § Department of Food and Drug, Faculty of Pharmaceutical Sciences, Federal University of Alfenas, Alfenas, Minas Gerais 37130-001, Brazil; ∥ Respiratory Technology, Woolcock Institute of Medical Research, Sydney, New South Wales 2037, Australia; ⊥ Faculty of Medicine and Health Sciences, Macquarie Medical School, Macquarie University, Sydney, New South Wales 2109, Australia

## Abstract

Paclitaxel (PTX) is a potent antiglioma agent, but its clinical use is limited by poor penetration across the blood–brain barrier (BBB). This study developed and evaluated a nose-to-brain delivery strategy using paclitaxel-loaded hybrid nanoparticles (HNP–PTX) for intranasal administration. A full 2^2^ factorial design was employed to optimize the formulation, yielding an HNP–PTX system with a monodisperse distribution (<200 nm) and spherical morphology. The impact of chitosan coating was evaluated, revealing changes in surface charge and spray properties; but this did not significantly enhance transport across RPMI-2650 cells cultured at the air–liquid interface. Importantly, all HNP–PTX formulations preserved nasal epithelial barrier integrity while significantly increasing PTX permeation relative to the free drug. Cytotoxicity assays in U87-MG glioblastoma cells showed that HNP–PTX reduced cell viability more than both taxol and temozolomide (TMZ). These *in vitro* findings suggest HNP–PTX is a promising candidate for further *in vivo* evaluation as an intranasal formulation for glioblastoma treatment.

## Graphical abstract

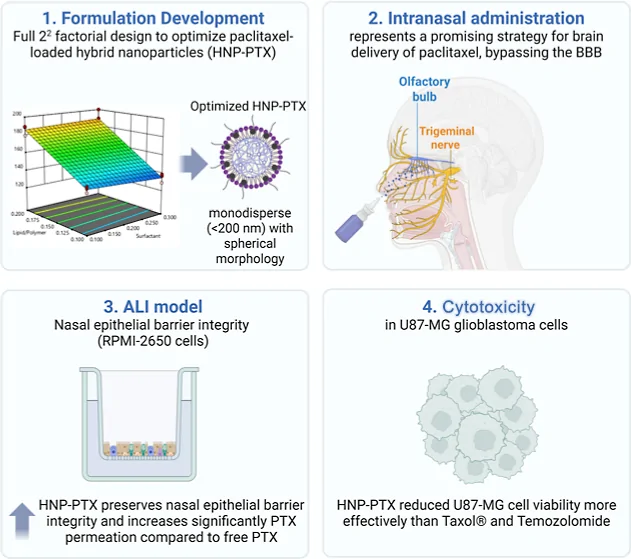

## 1 Introduction

The blood–brain barrier plays a crucial protective role but presents a significant challenge in delivering drugs to the central nervous system (CNS), hindering the treatment of neurological diseases, including brain tumors.

Paclitaxel, a well-established chemotherapeutic agent for solid tumors, is under investigation for its potential in treating brain tumors.^1,2^ According to the Cancer Cell Line Encyclopedia (CCLE), PTX is ∼1400-fold more potent than Temozolomide, the standard chemotherapeutic for brain cancer treatment.^3^ However, its clinical use has been limited by inadequate BBB penetration. Both intravenous PTX formulations (taxol and abraxane), although approved clinically, fail to cross the BBB. *In vitro* and *in vivo* studies have identified P-glycoprotein as a significant obstacle to PTX brain entry.^1,4^ Recent studies show that PTX-loaded nanocarriers can traverse a disrupted BBB and exert therapeutic effects in preclinical glioblastoma (GBM) models following intravenous administration.^5–8^ On the other hand, clinical trials with PTX covalently bound to poly-l-glutamic acid nanoparticles for GBM reported severe hematologic toxicity (grade 4 myelosuppression) without improved progression-free survival.^9,10^ These findings highlight the urgent need for novel therapeutic approaches to overcome BBB limitations and to optimize delivery systems that balance efficacy with safety.

Poly­(d, l-lactic-*co*-glycolic acid) (PLGA), a Food and Drug Administration (FDA)-approved biomaterial, has been used in nanoparticles to deliver PTX, either as a single drug or in combination with other agents.^11^ Combining materials with distinct chemical compositions can yield hybrid systems with properties superior to single-material formulations. In particular, the production of lipid-polymer hybrid nanoparticles (LPHN), achieved through the integration of organic compounds (e.g., phospholipids, proteins, lipids) with natural or synthetic polymers may result in systems with superior biocompatibility and enhanced system stability.^12^ While LPHN have been widely investigated for cancer drug delivery, relatively few studies have explored their application in glioblastoma, particularly via intranasal administration.

In this study, we developed LPHN composed of PLGA and distearoyl-*sn*-glycero-3-phosphoethanolamine–poly­(ethylene glycol) (DSPE–PEG) for PTX delivery (HNP–PTX) as a candidate system for intranasal administration. The intranasal route represents a promising, noninvasive approach to bypass the BBB, offering direct access to the brain.^13^ Drugs administered intranasally can access the brain through neuronal pathways, including the olfactory and trigeminal nerves, and subsequently distribute through cerebrospinal fluid. This pathway can improve drug bioavailability in the CNS while reducing systemic clearance and degradation, making it especially attractive for CNS-active agents with limited BBB permeability.^13,14^

Accordingly, unloaded hybrid nanoparticles (HNP) were first prepared using PLGA. A 2^2^ full factorial experimental design was applied to optimize lipid/polymer ratios and surfactant concentrations, with particle diameter, polydispersity index (PDI), and surface charge as response variables. Optimized HNPs were subsequently loaded with PTX, and the effects of drug incorporation on physicochemical properties were assessed. *In vitro* transport studies assessed PTX permeation from different HNP–PTX formulations across RPMI-2650 cells cultured under an air–liquid interface (ALI) model. Following application of the developed formulation, transepithelial electrical resistance (TEER) measurements and paracellular permeability assays were performed to evaluate barrier integrity. Finally, HNP–PTX cytotoxicity was evaluated in human glioblastoma U87-MG cells and nasal epithelial RPMI-2650 cells. This study, to our knowledge, is among the first to combine factorial optimization, spray device testing, and ALI transport models for PTX-loaded HNPs designed for nose-to-brain delivery.

## 2 Materials

### 2.1 Chemicals

DSPE–PEG_2000_ was purchased from Lipoid GmbH (Ludwigshafen, Germany). PLGA was obtained from Boehringer Ingelheim (Ingelheim am Rhein, Germany). Polysorbate 80 was provided by Croda do Brasil Ltd. (Campinas, Brazil). PTX was provided by Quiral Química do Brasil S/A (Juiz de Fora, Brazil) with a purity greater than 97%. Taxol was obtained from Accord Farmacêutica Ltd. (São Paulo, Brazil). Acetone was purchased from Êxodo Científica (São Paulo, Brazil), and HPLC-grade acetonitrile from Sigma-Aldrich (St. Louis, MO, USA).

### 2.2 Cells

Human glioblastoma U87-MG cells were purchased from the American Type Culture Collection (ATCC). Dulbecco’s modified Eagle’s medium (DMEM), trypsin, tris­(hydroxymethyl)­aminomethane (Tri’s base), and sulforhodamine B (SRB) were obtained from Sigma-Aldrich (St. Louis, MO, USA). Fetal bovine serum (FBS) and penicillin/streptomycin/amphotericin (PSA) were sourced from Gibco Life Technologies (Carlsbad, CA, USA). All cell lines were routinely screened for mycoplasma contamination by polymerase chain reaction (PCR), with negative results.

## 3 Experimental Section

### 3.1 Optimization of Hybrid Nanoparticle Preparation Using an Experimental Factorial Design

A 2^2^ full factorial design was applied to optimize nanoparticle preparation, with lipid/polymer ratio and surfactant concentration as independent variables at high (+1) and low (−1) levels, as seen in Table 1. PLGA (50:50 lactide/glycolide) was tested. Four formulations were prepared in triplicate. Dependent variables were average diameter and zeta potential. Statistical analysis was performed using Stat-Ease 360 Software (version 22.0, Minneapolis, MN, USA) at *p* ≤ 0.05.

**Table 1:** Levels of Independent Variables in the 2^2^ Factorial Design

	levels	
independent variable	–1	+1
surfactant concentration (%)	0.1	0.3
polymer/lipid proportion (w/w)	0.1	0.2

### 3.2 Nanoparticle Preparation

#### 3.2.1 Unloaded Hybrid Nanoparticle

Unloaded HNPs were prepared by nanoprecipitation followed by solvent evaporation.^15^ An organic phase containing PLGA (50:50), DSPE–PEG_2000_, and polysorbate 80 (w/v) in acetone (2.0 mL) was poured into 6 mL of aqueous phase (4% ethanol) under agitation. The mixture was stirred at 600 rpm for at least 12 h on a magnetic stirrer to evaporate the organic solvent under atmospheric pressure.

#### 3.2.2 HNP–PTX

HNP–PTX were prepared as above with a lipid/polymer ratio (DSPE–PEG/PLGA 50:50) of 0.1% and surfactant 0.1% (w/v). Briefly, PTX (0.5 mg/mL in acetone) was added to the organic solution. Free PTX was removed by ultrafiltration/centrifugation using Amicon Ultra-15 centrifuge filters (100 kDa; Amicon, Danvers, USA) at 4000 rpm for 5 min, followed by three washes with ethanol.

#### 3.2.3 Chitosan Coating of Nanoparticles

To evaluate the influence of surface charge on HNP–PTX behavior *in vitro*, purified nanoparticles were coated with low-viscosity chitosan (CS, 50–190 kDa, viscosity 20–300 cP, deacetylation degree > 75%) at different concentrations. CS solutions were prepared by dissolving CS powder in 1% (v/v) aqueous acetic acid. Aliquots of CS solution were then added dropwise to the HNP dispersion (final CS 5–250 μg/mL) under constant stirring for 15 min. CS-coated formulations were designated HNP/CS, using a 1:1 (*v*/*v*) mixing ratio.

### 3.3 Nanoparticle Characterization

#### 3.3.1 Average Diameter, Polydispersity Index, and Zeta Potential Measurements

Average diameter and PDI were determined by dynamic light scattering (DLS) at 25 °C and a 90° (unimodal analysis). Zeta potential was evaluated by electrophoretic mobility at 90° and 25 °C after 100-fold dilution in an aqueous solution of 1 mmol/L NaCl (w/v), previously filtered through 0.45 μm. Measurements were performed on at least three independent batches, each in triplicate, using a Zetasizer Nano ZS90 (Malvern Instruments Ltd., Malvern, England). Data are expressed as mean ± standard deviation (SD).

#### 3.3.2 Encapsulation Efficiency

Encapsulated PTX was quantified by high-performance liquid chromatography (HPLC) following the same protocol and conditions described. The chromatographic conditions were an eluent system consisting of acetonitrile: water (55:45, *v*/*v*) at a flow rate of 1.2 mL/min, ambient temperature, and an injection volume of 20 μL. Analyses used an Agilent 1260 Infinity instrument (Santa Clara, CA, USA) with a G4212B diode-array detector (Santa Clara, CA, USA) and a Lichrospher C18 reverse-phase column (5 μm, 4 mm × 25 cm; Merck Millipore, Darmstadt, Germany). Detection was performed at 254 nm.^16^ Encapsulation efficiency (EE) was calculated according to eq 1

1 %EE=(druginpurifiednanoparticles/druginnon‐purifiednanoparticles)×100

#### 3.3.3 Nanoparticle Tracking Analysis

Particle concentration and size distribution were measured by nanoparticle tracking analysis (NTA) using a NanoSight NS300 (Malvern Panalytical Ltd., Malvern, United Kingdom). Samples were diluted in ultrapure water. For each sample, five analyses were performed, with 60 s videos recorded at 25 °C. NTA 3.3 software was used to analyze particle concentration. Data are expressed as mean ± standard deviation (SD) for at least three batches per formulation.

#### 3.3.4 Evaluation of Thermal Properties

The thermal behavior of pharmaceutical ingredients and nanoparticle formulations was evaluated by differential scanning calorimetry (DSC) and thermogravimetric analysis (TG). DSC was performed on a DSC60 Shimadzu (indium calibrated) under dynamic nitrogen atmosphere (50 mL/min) with a heating rate of 10 °C/min range 30–400 °C using sealed aluminum crucible (1.5 mg, accurately weighed).

For TG analyses, the curves were obtained using a Shimadzu DTG60H thermobalance in a dynamic nitrogen atmosphere (50 mL/min), heating rate of 10 °C/min, over 30–500 °C. An open alumina crucible and a sample mass of approximately 2.0 mg were employed. Afterward, the first-order derivative of the TG curves (DTG) was calculated to evaluate the mass loss, temperature of thermal phenomena, proportional to the amount of substance remaining.^17–19^

#### 3.3.5 Transmission Electron Microscopy

Formulations were assessed for morphology by transmission electron microscopy (TEM). Samples were diluted 1:200 in ultrapure water, and 3 μL of each was placed onto copper grids covered with Formvar film. Grids were stained with aqueous phosphotungstic acid solution (1% v/v), air-dried, and imaged using Tecnai G2–12 Spirit BioTWIN microscope (FEI/Philips, The Netherlands) operated at 120 kV (Quanta platform).

### 3.4 Spray and Aerosol Characterization of Formulations

HNP–PTX formulations, including HNP–PTX–CS at different concentrations, were loaded into a VP7 Aptar nasal valve to evaluate spray performance.

#### 3.4.1 Laser Diffraction Droplet Sizing

Droplet-size distribution was assessed using a Malvern Spraytec laser diffraction system (Malvern Instruments, Malvern, Worcestershire, UK). Experiments were conducted at 25 °C with a 30° actuation angle, and the device was positioned 2.5 cm from the measurement cell. Volume distributions were expressed as Dv10, Dv50, and Dv90 percentiles, and span was calculated as (Dv90–Dv10)/Dv50.

#### 3.4.2 Deposition Profiles Using a Transparent Silicone Human Nose Model

Deposition profiles were measured in a Koken transparent silicone human nose model. This static nasal model allows visualization of nasal deposition but does not simulate mucociliary clearance or airflow, limiting its ability to predict *in vivo* drug distribution. The inner surface, including the nasopharynx, was uniformly coated with approximately 0.5 g of Sar-Gel. Before each test, the model was conditioned in a sealed box with silica gel and equilibrated at 37 °C for 20 min. For actuation, a VP7 Aptar nasal spray pump was inserted 5 mm into the nostril of the vertically positioned model and actuated at a 45° angle. Images were captured every 5 min for 20 min with the model maintained at 37 °C, then analyzed with ImageJ software. Coverage of the nasal cavity and olfactory region was defined as the percentage of the respective areas showing color change. The olfactory region was identified based on established CT-based nasal segmentation methods.

### 3.5 *In Vitro* Biological Characterization of the PTX Formulations

#### 3.5.1 Air–Liquid Interface Model

The RPMI-2650 human nasal epithelial cell line, obtained from the ATCC, was used for biological characterization of the nasal formulations. Cells (passages 30 and 45) were cultured at 37 °C in a humidified atmosphere containing 5% CO_2_. The culture medium was minimum essential medium (MEM; Gibco, USA) supplemented with 10% (v/v) heat-inactivated fetal bovine serum (Gibco, Australia) and 1% (v/v) nonessential amino acids (Sigma-Aldrich). To establish the ALI model, RPMI-2650 cells were seeded onto the apical surface of Snapwell inserts (Corning Costar, polyester membrane, 0.4 μm pore size, 12 mm diameter) at 2.5 × 10^6^ cells/mL in 6-well plates. Cells were initially cultured under submerged conditions with 2 mL of complete medium in the basolateral chamber. After 2 days, the apical medium was aspirated, and basolateral medium was replaced every other day to promote differentiation under ALI conditions for 14 days.

#### 3.5.2 *In Vitro* Drug Transport Studies

To assess PTX transport from free and HNP–PTX formulations across ALI-cultured RPMI-2650 monolayers, inserts were equilibrated at 37 °C. Formulations were deposited onto the cells using a modified nasal expansion chamber connected to the next generation impactor (NGI, Westech W7; Westech Scientific Instruments, UK).^20^ The formulations were actuated with a VP7 Aptar nasal device. For each experiment, five actuations of formulations containing PTX at 100 μg/mL were applied, with each actuation delivering approximately 100 μL, resulting in a total administered volume of approximately 500 μL (50 μg PTX). Snapwell inserts were then transferred to 6-well plates containing 2 mL of prewarmed Hanks’ balanced salt solution (HBSS) in the basolateral compartment and incubated at 37 °C for 4 h. Basolateral samples (200 μL) were collected every 30 min and replaced with fresh HBSS. PTX levels were quantified using HPLC. Residual apical drug was assessed by washing the inset surface with HBSS, and intracellular PTX content was determined by lysing cells with CelLytic buffer (Sigma-Aldrich).

#### 3.5.3 Transepithelial Electrical Resistance

TEER measurements were performed to evaluate epithelial integrity using an EVOM2 epithelial volt/Ω meter (World Precision Instruments, USA) with chopstick electrodes. As described in the previous section, the formulations were administered using five actuations of the nasal spray, resulting in a total PTX dose of 50 μg. Resistance values were corrected for the background of the Snapwell insert. Before drug application, 0.3 mL HBSS was added apically and 2 mL basolaterally; apical HBSS was then removed to restore ALI conditions. After 4 h of exposure, TEER values were measured again.

#### 3.5.4 Paracellular Permeability Assessment

To assess effects of HNP–PTX on tight junctions and paracellular transport, sodium fluorescein (Flu-Na) was used as a paracellular marker. After the 4 h transport study, cells were gently rinsed with HBSS to remove residual formulation. Then, 200 μL of Flu-Na solution (2.5 mg/mL in HBSS) was added apically and incubated for 1 h at 37 °C. Basolateral samples (200 μL) were collected every 15 min and replaced with fresh HBSS. Fluorescence intensity was measured on a SpectraMax M2 reader (excitation 485 nm, emission 535 nm). The apparent permeability coefficient (*P*_app_) was calculated using the formula eq 2

2 $$P_{app}=\left(dQ/dt\right)/\left(C_{0}\times A\right)$$

where d*Q*/d*t* is the mass flux of Flu-Na (μg/s), *C*_0_ is the initial donor concentration (μg/mL), and *A* is the surface area of the cell monolayer (cm^2^).

### 3.6 Cytotoxicity Study against Glioblastoma and Nasal Epithelial Cells

Human glioblastoma U87-MG cells were cultured in DMEM medium supplemented with 10% (v/v) FBS, 100 IU/mL penicillin, and 100 μg/mL streptomycin at 37 °C, 5% CO_2_. Cells were seeded at 1 × 10^4^ cells/well in 96-well plates and incubated for 24 h, then treated for 48 h with (1) HNP–PTX (7.5 μM to 0.096 nM), (2) TMZ solution (2400 μM to 30.72 nM), (3) Taxol (7.5 μM to 0.096 nM), (4) unloaded HNP, and 20% dimethyl sulfoxide (DMSO) as a positive control. Following treatment, cells were fixed and cytotoxicity was assessed using the sulforhodamine B colorimetric assay.^21^

RPMI-2650 nasal epithelial cells were cultured in RPMI-minimum essential medium supplemented with 10% FBS at 37 °C, 5% CO_2_. Cells were seeded in 96-well plates at 5 × 10^6^ cells per well and incubated overnight for adherence. HNP–PTX and free PTX were diluted in medium to achieve final concentrations of 100–0.39 μM. Cell viability was assessed using the MTS assay (CellTiter 96 Aqueous One Solution Cell Proliferation Assay, Promega) according to the manufacturer’s protocol. After 48 h of treatment exposure, MTS reagent was added, and plates were incubated at 37 °C for 2 h. Absorbance was measured at 490 nm.

The choice of different assays was based on cell characteristics. For U87 cell line, which is highly resistant and capable of activating various survival mechanisms, the SRB assay is advantageous because it measures total cellular protein and is less affected by transient metabolic fluctuations.^22,23^ In contrast, since RPMI-2650 cells are fast-growing and metabolically active, the mitochondrial activity assessment through the MTS assay is a more immediate and informative approach for evaluating the effects of cytotoxic compounds.^24^ For both cell lines, each condition was tested in triplicate (*n* = 3). Cell viability was calculated as a percentage relative to untreated controls. The half-maximal inhibitory concentration (IC_50_) values were calculated using GraphPad Prism 8.0.2 (GraphPad Software, La Jolla, CA, USA).

### 3.7 Statistical Analysis

All data are expressed as mean ± standard deviation. Normality and homoscedasticity of variance were assessed using the Kolmogorov–Smirnov and Brown–Forysthe tests, respectively. Data were analyzed using analysis of variance (ANOVA) followed by Tukey’s post hoc test. For comparisons between two groups, Student’s *t*-test with residual analysis was used where appropriate. Statistical analyses were conducted in GraphPad Prism (version 8.0.2, La Jolla, CA, USA). A 95% confidence interval was adopted, and differences were considered significant at *p* ≤ 0.05.

## 4 Results and Discussion

### 4.1 Investigating the Influence of Lipid/Polymer Ratio and Surfactant Concentration on Hybrid Nanoparticles

Four combinations of the high and low levels of the independent variables were prepared. Each formulation was prepared in triplicate (12 total runs). Replication allowed estimation of experimental error for each response and, subsequently, assessment of whether the factors exerted significant effects. The effects of the independent variables on the dependent variables (diameter and zeta potential) were investigated, and the results are presented in Table 2.

**Table 2:** Diameter and Zeta Potential of Nanoparticles Prepared Under Different Experimental Arrangements^a^

lipid/polymer	surfactant	diameter (nm)	zeta potential (mV)
**–1**	**–1**	140.6 ± 2.7	–30.7 ± 2.5
**–1**	**+1**	134.7 ± 4.2	–29.5 ± 2.3
**+1**	**–1**	185.8 ± 4.5	–36.4 ± 3.1
**+1**	**+1**	188.8 ± 9.3	–35.1 ± 2.2

a Data are presented as mean ± standard deviation of three independent experiments.

Analysis of variance indicated that the models were significant for both diameter and zeta potential for PLGA 50:50 (*p* < 0.05). The lipid/polymer ratio was a significant factor, strongly influencing diameter and zeta potential (*p* < 0.0001 and *p* = 0.0023, respectively), whereas increasing surfactant concentration did not significantly affect these responses (Figure 1). The coded mathematical models were given by eqs 3 and 4

3 $$Y_{1}\left(diameter\right)=162.46-{0.72X}_{1}+{24.82X}_{2}+{2.21X}_{1}X_{2}$$

4 $$Y_{2}\left(zeta\;potential\right)=-32.05+{0.012X}_{1}-{2.70X}_{2}+{0.053X}_{1}X_{2}$$

where *X*_1_ is surfactant concentration, *X*_2_ is lipid/polymer ratio, and *X*_1_ *X*_2_ is the interaction between the independent variables. A difference <0.2 between predicted and adjusted *R* ^2^ for diameter indicated good agreement. Adequate precision values > 4.0 further supported reliable navigation of the design space (Figure 1).

**Figure 1:**
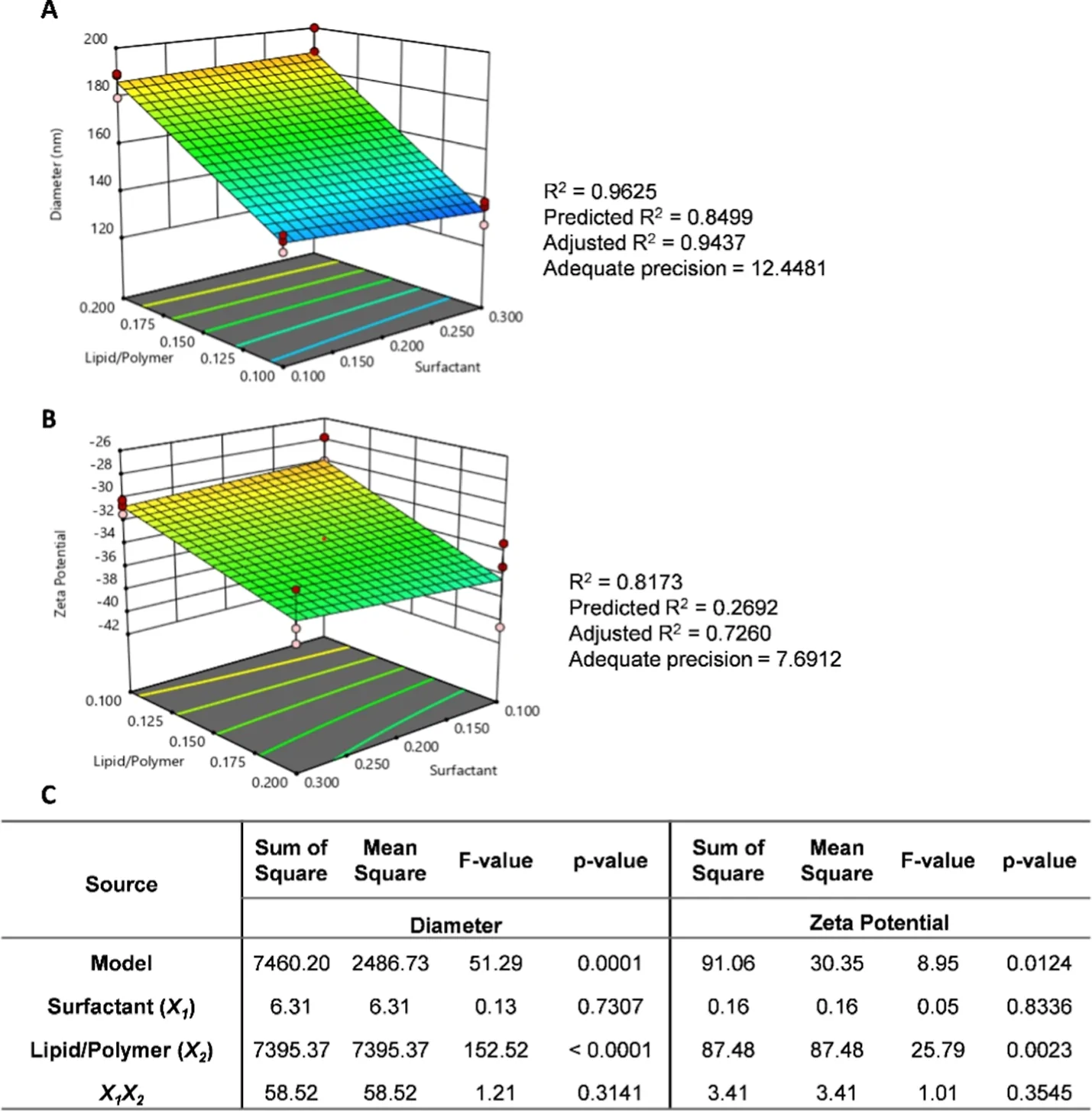
Model summary derived from a 2^2^ full factorial design. Three-dimensional response surface plots illustrating the effects of the lipid/polymer ratio and surfactant concentration on nanoparticle diameter (A) and zeta potential (B). ANOVA outputs indicating factor significance are also shown (C).

Considering that all formulations exhibited a mean particle diameter below 200 nm, which favors diffusion through nasal mucus, the first barrier to intranasal drug transport, as well as zeta potential values indicative of highly charged surfaces that help prevent aggregation and coalescence over time, the HNP formulation containing the lowest levels of surfactant and polymer/lipid was selected as the optimized formulation, as higher concentrations of these components did not provide any additional advantage in terms of particle size or zeta potential.^25–30^

The accuracy of the model was validated by conducting three parallel experiments using the predicted optimal formulation. The mean particle size was 146.3 ± 5.7 nm (predicted 140.6 nm) and the mean of zeta potential was −35.7 ± 0.5 mV (predicted −29.9 mV). The experimental results confirmed the adequacy of the optimization model. The particle size showed excellent agreement with the predicted value. On the other hand, a larger deviation was observed in the zeta potential, likely due to the lower predictive capability of the model for this response (predicted *R* ^2^ = 0.27). However, this response was still satisfactorily described by the model, as indicated by the adjusted *R* ^2^ (0.7260) and adequate precision (7.6912) values. It is worth noting that the experimental zeta potential remained highly negative, confirming that the optimized formulation exhibited the desired physicochemical characteristics.

### 4.2 Characterization of HNP–PTX

To evaluate the PTX loading capacity, HNPs with a lipid/polymer ratio of 0.1 were prepared using PTX at 0.5 mg/mL and varying surfactant concentrations (0.1, 0.2, and 0.3% w/v). To evaluate the PTX loading capacity, HNPs with a polymer/lipid proportion of 0.1 were prepared using PTX at 0.5 mg/mL and surfactant concentrations of 0.1, 0.2, and 0.3% (w/v). This formulation was selected because its predicted particle size provided a greater safety margin for maintaining nanoparticle diameters below the target limit of 200 nm after PTX encapsulation, which is expected to increase particle size, while preserving the desired physicochemical characteristics.

As shown in Figure 2, increasing surfactant concentration resulted in a significant decrease in encapsulated PTX concentration (*p* < 0.0001). Encapsulation efficiency showed the same trend (Figure 2), suggesting that higher surfactant levels may compromise drug entrapment, possibly by enhancing PTX solubilization in the external phase during nanoparticle formation.^31,32^

**Figure 2:**
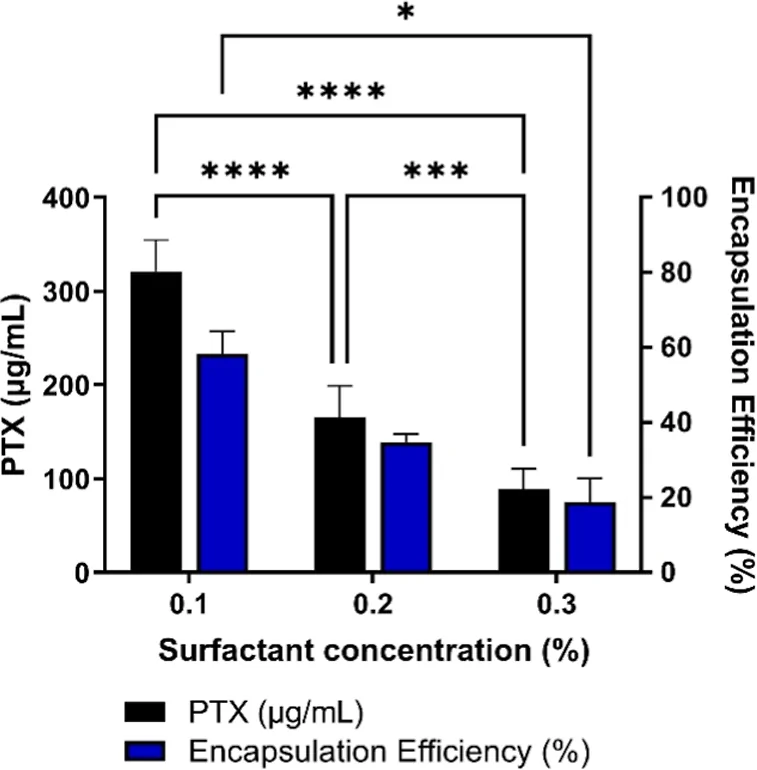
Variation in encapsulation efficiency and drug concentration of PTX-loaded hybrid nanoparticles as a function of surfactant concentration. Data are presented as mean ± standard deviation of at least three independent experiments. *, ***, and **** denote *p* < 0.05, 0.001, and 0.0001, respectively.

Thus, the formulation using the lowest lipid/polymer ratio (equivalent to 1 mg DSPE–PEG_2000_ and 10 mg of PLGA), the lowest surfactant concentration (0.1% w/v), and PTX 0.5 mg/mL, referred to as HNP–PTX, was selected for further characterization.

A PDI of 0.095 ± 0.021 for HNP and 0.098 ± 0.021 for HNP–PTX indicates a monodisperse nanosystem. Furthermore, these values align with typical acceptable criteria for polymer- and lipid-based nanoparticles, for which PDI <0.2 and <0.3, respectively, suggesting the monodispersity of the system.^33,34^

Zeta potentials of −33.2 ± 0.2 mV (HNP) and −31.9 ± 6.6 mV (HNP–PTX) indicate highly charged surfaces that help prevent aggregation and coalescence over time, suggesting good physical stability of the system. Similar zeta potentials (more negative than −25 mV) have been reported for PLGA nanoparticles, often attributed to surface carboxylic groups.^29,30^

The average diameter of HNP–PTX was evaluated by DLS and NTA techniques (Table 3). Nanostructures with average diameters smaller than 200 nm can diffuse through nasal mucus, which is the first barrier to intranasal drug transport, and may favor retention in tumor tissue.^25–28^ NTA technique demonstrated that 90% of nanoparticles were <200 nm, while DLS yielded D90 ≈ 240 nm. The difference arises from methodology principles: NTA derives the size of liquid suspension from Brownian motion and light scattering/refraction. This method can detect both small and large particles with high resolution, thereby providing a more precise particle distribution. The particle concentrations determined by NTA method were 5.3 ± 0.5 × 10^14^ particles/mL for HNP and 1.9 ± 0.9 × 10^13^ particles/mL for HNP–PTX.

**Table 3:** HNP–PTX Average Diameter Obtained from DLS and NTA Analyses^a^

	DLS	NTA
diameter (nm)	171.8 ± 1.2	145.3 ± 1.2
D10	117.6 ± 3.0	105.0 ± 1.4
D50	159.0 ± 2.2	135.3 ± 0.5
D90	240.2 ± 4.5	199.0 ± 7.3

a Data are presented as mean ± standard deviation of three independent experiments.

To analyze nanoparticle morphology, TEM was used. Nanoparticles produced with and without PTX exhibited a core–shell structure, well-defined spherical shape, and diameters <200 nm (Figure 3). Similar features were previously reported for PLGA-based hybrid nanoparticles,^35–37^ in agreement with the present findings. Furthermore, those authors proposed that the dark outer layer corresponds to a lipid shell surrounding a bright PLGA core. The absence of bright regions in the shell suggests that PLGA is concentrated within the core.^37,38^

**Figure 3:**
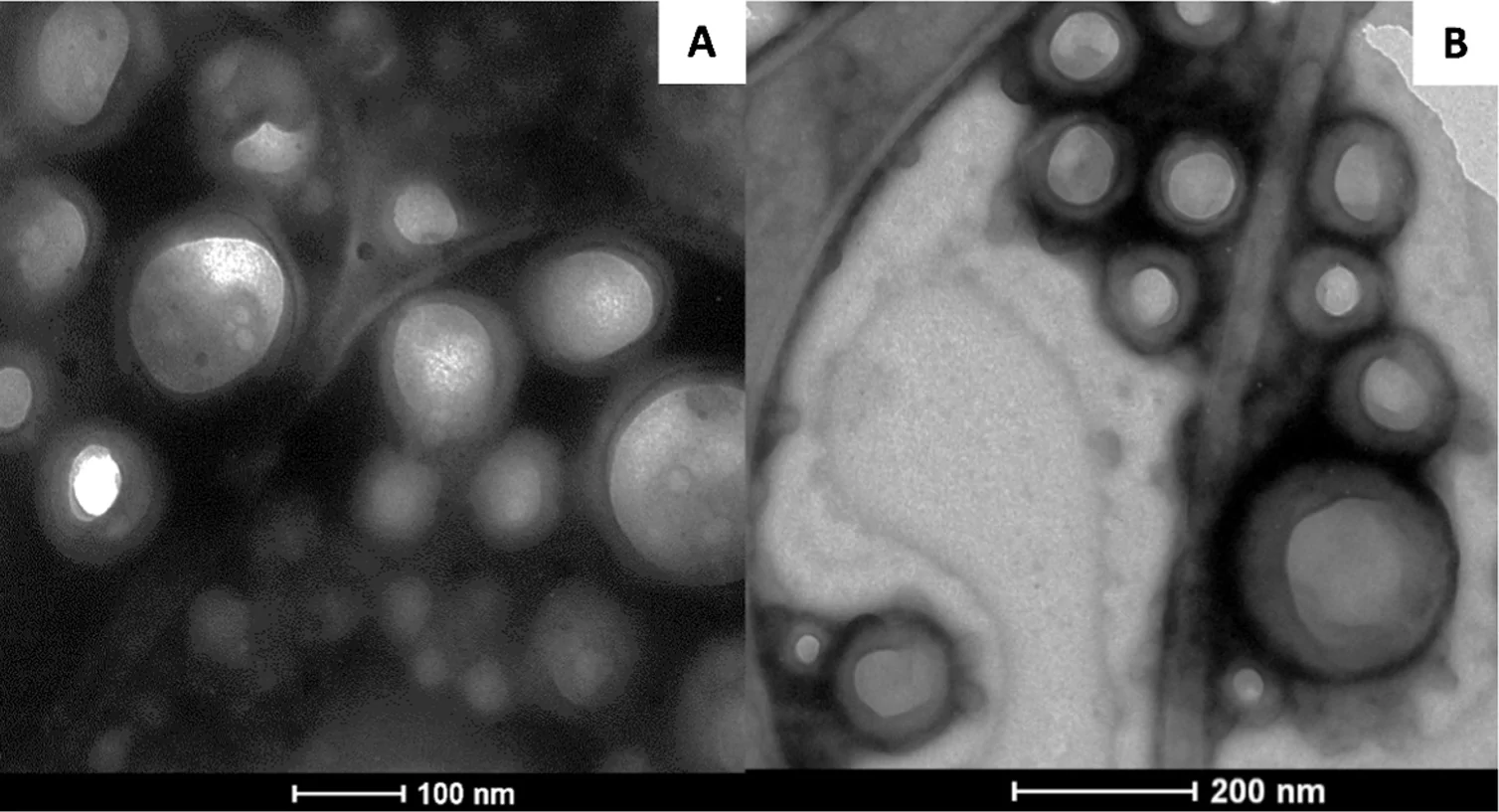
Transmission electron microscopy images of (A) HNP and (B) HNP–PTX.

### 4.3 Evaluation of Thermal Properties

Thermal analysis of the individual formulation compounds was conducted using DSC and TG to investigate possible interactions (Figure 4). The DSC curve of PTX exhibited an endothermic peak at 212.58 °C (*T*_onset_), with an enthalpy change of 50.06 J g^–1^, corresponding to its melting point. An exothermic event associated with PTX degradation (shaded rectangle in Figure 4A) indicates the thermal stability up to 216 °C was also observed and confirmed by TG curve. For DSPE–PEG_2000_, an endothermic event around 50 °C, characteristic of melting, was observed, similar to that reported by Maia (2022) and Tahir et al. (2019).^37^ For PLGA, a baseline change was observed between 25 and 55 °C corresponding to the glass transition (*T*_g_), followed by a peak related to polymer melting (Figure 4A, shaded circle). An additional endothermic peak around 350 °C indicated polymer decomposition, which was confirmed by the TG curve. TG data (Figure 4B) revealed that PLGA maintained thermal stability up to approximately 215 °C, after which decomposition began. A mass loss of nearly 100% occurred in a single step, between 225 and 375 °C, consistent with a decomposition process previously described by Silva et al. (2015).^39^ DSPE–PEG_2000_ and Tween 80 also showed single-step decomposition, with mass loss occurring up to approximately 425 °C.

**Figure 4:**
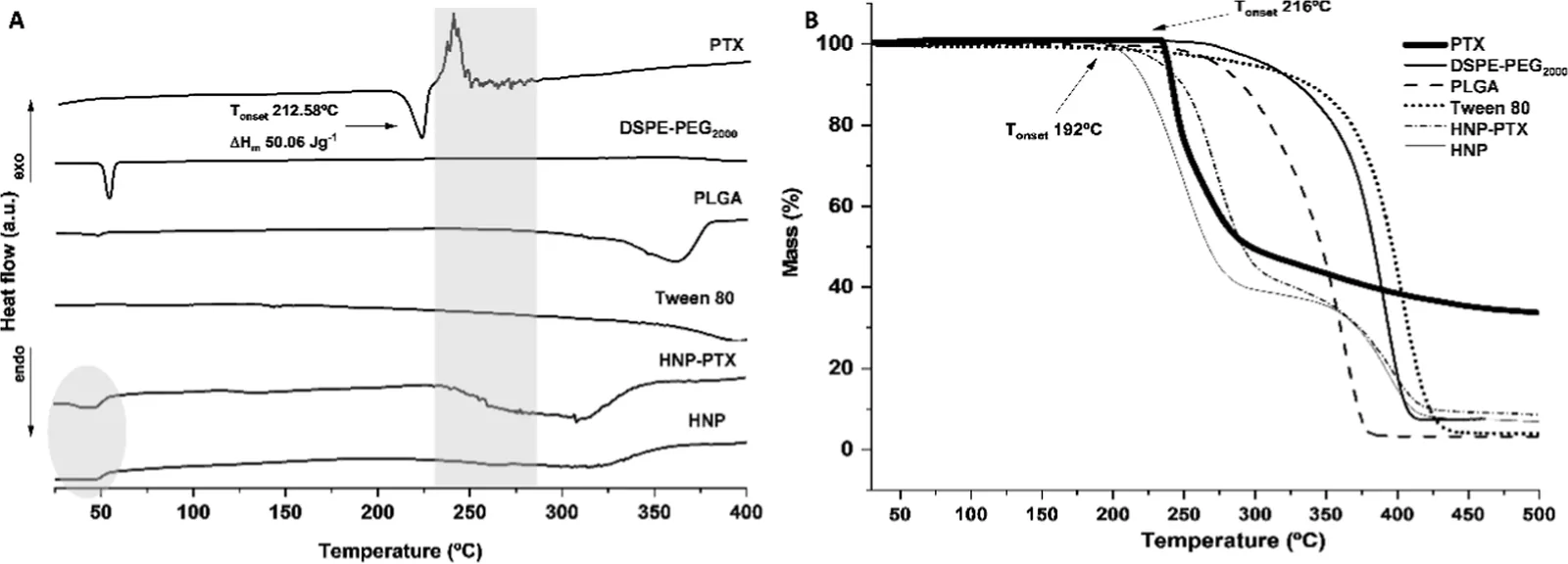
Thermal analysis of samples. (A) Differential scanning calorimetry curves showing heat flow and (B) thermogravimetric curves showing thermal stability of PTX, DSPE–PEG_2000_, PLGA, Tween 80, HNP, and HNP–PTX.

Regarding HNP–PTX, no melting of the drug is observed, suggesting that PTX is encapsulated.^40^ Additionally, no significant changes in thermal behavior were observed compared with the individual compounds, indicating adequate compatibility. The blank and PTX-loaded nanoparticles exhibit an endothermic phenomenon between 30 and 60 °C, as indicated by the shaded circle in Figure 4A (no mass loss in the TG curve), related to the melting of the lipid phase. TG analysis showed a two-step decomposition for both HNP and HNP–PTX. The first step occurred between 192 and 300 °C, and the second step started around 350 °C and finished at around 425 °C. Nanoparticles containing PTX exhibit superior thermal stability compared to the blank formulation, with favorable behavior for the manufacturing process (Figure 4B).

### 4.4 Chitosan Coating of Nanoparticles

To assess the influence of surface charge on the *in vitro* behavior of HNP–PTX, nanoparticles were coated with low-viscosity chitosan at various concentrations (5–250 μg/mL) after purification. Chitosan was selected as a mucoadhesive polymer because it is known to reversibly open tight junctions, thereby improving intranasal drug permeation.^41^

As shown in Figure 5A, particle size increased with CS concentration up to 50 μg/mL and subsequently decreased at higher CS concentrations (125 and 250 μg/mL). The initial increase in particle size is consistent with CS deposition on the nanoparticle surface through electrostatic attraction between the cationic CS and the negatively charged lipid-polymer hybrid nanoparticles. At intermediate CS concentrations (25 and 50 μg/mL), the relatively low absolute zeta potential may have provided insufficient electrostatic stabilization, promoting particle aggregation and resulting in larger hydrodynamic diameters. As the CS concentration increased further, the higher positive zeta potential enhanced electrostatic repulsion between particles, reducing aggregation and consequently decreasing particle size.

**Figure 5:**
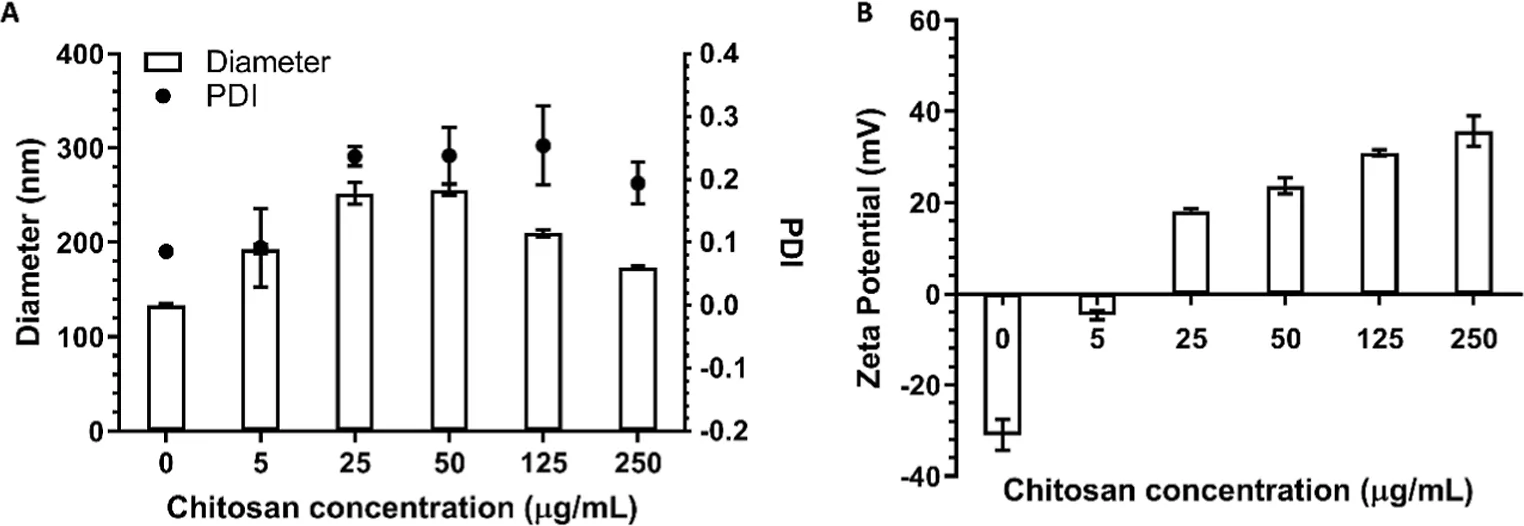
Effect of CS concentration on diameter and PDI (A) and zeta potential (B) of PTX-loaded NP during the coating process. Each CS solution was mixed 1:1 (v/v) with the HNP–PTX dispersion. Data are expressed as the mean ± standard deviation (*n* = 3).

Zeta potential shifted progressively toward more positive values as CS concentration increased (Figure 5B), consistent with adsorption of cationic CS on the nanoparticle surface. Based on these results, three formulations were selected for further evaluation: uncoated HNP–PTX (negative charge, −31.9 ± 6.6 mV), HNP–PTX-CS5 (near-neutral charge, −4.7 ± 0.7 mV), and HNP–PTX-CS250 (positive charge, +35.7 ± 2.3 mV).

### 4.5 Spray Pattern Characterization and Nasal Deposition Pattern

Formulations were loaded into a VP7 Aptar nasal valve, and spray performance was assessed as Dv10, Dv50, Dv90, and span using the Spraytec laser diffraction system. Spray performance of nasal formulation is a critical parameter of deposition and, per FDA expectations, should be thoroughly characterized. It is governed primarily by device design and actuation conditions, as well as formulation properties.^42^

Across HNP–PTX formulations delivered via the VP7 Aptar nasal valve, all three produced droplet sizes with Dv50 < 150 μm (Table 4), indicating suitability for nasal delivery. However, spray performance differed among formulations: the highly concentrated HNP–PTX-CS250 exhibited significantly larger Dv10, Dv50, and Dv90 than uncoated HNP–PTX. This shift can be attributed to formulation properties, particularly viscosity, which strongly influence nasal aerosolization and interact with device and actuation parameters. Increases in viscosity are known to raise Dv50, especially in polymer-based nasal formulations.^43^ Consistent with this, viscosity rose from 1.32 ± 0.05 mPa·s (uncoated HNP–PTX) to 1.69 ± 0.05 mPa·s (HNP–PTX-CS5) and 2.26 ± 0.01 mPa·s (HNP–PTX-CS250) at 37 °C. The higher viscosity of HNP–PTX-CS250 likely hindered atomization, resulting in larger droplets than the less viscous, uncoated formulation.

**Table 4:** Droplet Size Distribution of HNP–PTX Without or with CS Coating at Different Concentrations^a^

formulations	Dv10 (μm)	Dv50 (μm)	Dv90 (μm)	span
HNP–PTX	42.3 ± 0.9	118.6 ± 3.9	262.1 ± 9.1	1.85 ± 0.02
HNP–PTX CS5	51.8 ± 2.2	137.7 ± 4.2	314.0 ± 12.4*	1.84 ± 0.04
HNP–PTX CS250	57.3 ± 7.0*	147.4 ± 12.8*	324.0 ± 22.2*	1.78 ± 0.09

a Data are expressed as mean ± standard deviation (*n* = 3). *p < 0.05 vs HNP–PTX. Dv10, Dv50, and Dv90 denote the diameters below which 10%, 50%, or 90% of droplets fall.

Despite this effect on atomization, all formulations generated droplet sizes within the range considered appropriate for nasal delivery. Moreover, the larger droplet size of HNP–PTX-CS250 did not compromise deposition in the olfactory region. Distinct mechanisms govern aerosol generation and regional nasal deposition: whereas viscosity primarily affects droplet formation during atomization, nasal cavity deposition is also influenced by airflow patterns, spray plume characteristics, and nasal geometry.^44^ Consequently, the modest increase in droplet size observed for HNP–PTX-CS250 remained compatible with the olfactory region and exhibited significantly greater deposition than HNP–PTX, using a Koken transparent nasal cast (Figure 6). None of the formulations dripped into the throat, suggesting good retention within the nasal cavity. Enhanced olfactory deposition, together with the well-established mucoadhesive properties of chitosan, may provide an advantage for nose-to-brain drug delivery. Nevertheless, optimization of chitosan molecular weight and degree of deacetylation may further improve the balance between spray performance and biological functionality, as these parameters were not investigated in the present study. Furthermore, these *in vitro* deposition results provide only an approximation of *in vivo* performance.

**Figure 6:**
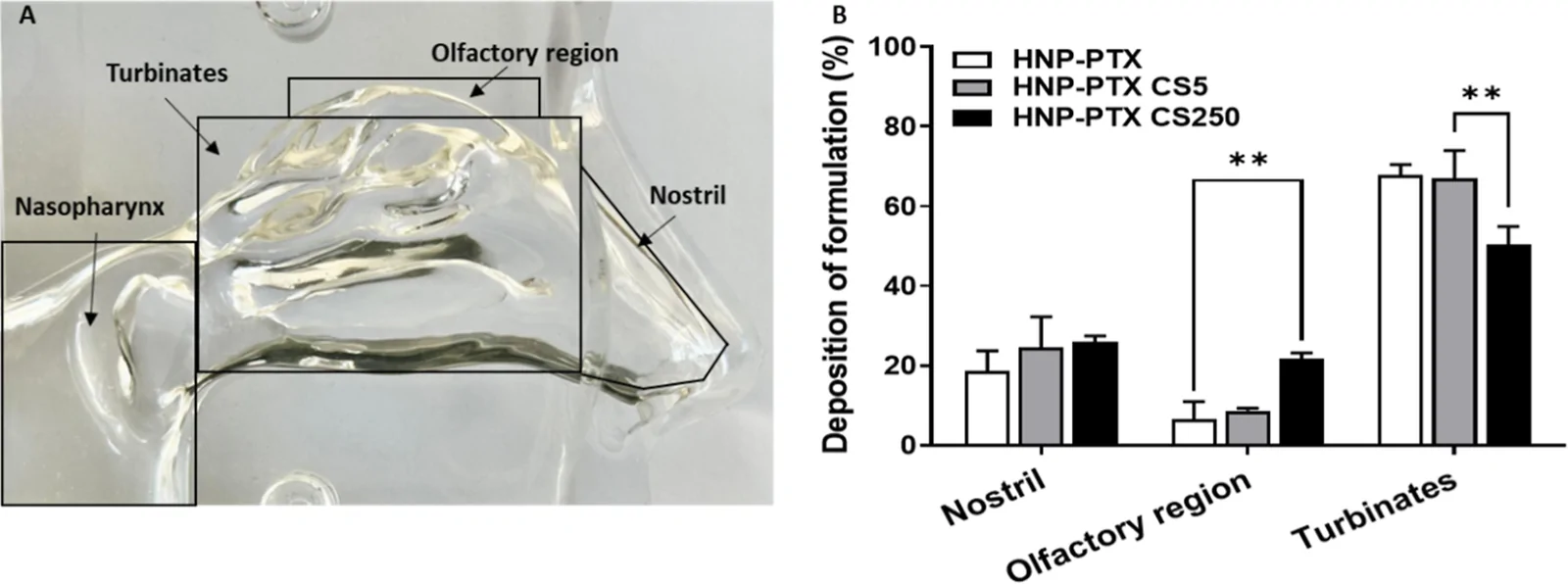
(A) Koken transparent nasal cast employed in the nasal deposition experiment and the regions identified for evaluating deposition. (B) Drug deposition fractions of HNP–PTX formulations across regions of the silicone nasal cavity model, evaluated immediately after aerosolization (0 min). *p < 0.05 vs HNP–PTX.

### 4.6 Evaluation of Nasal Epithelial Barrier Integrity via TEER and Paracellular Permeability

To determine whether HNP–PTX formulations disrupt the nasal epithelial barrier, a crucial consideration for nose-to-brain drug delivery, TEER values were measured. Baseline TEER (Ω·cm^2^) was recorded to confirm barrier integrity. After 4 h of exposure, none of the HNP–PTX formulations produced a significant change in TEER relative to baseline (*p* > 0.05). This finding suggests that the formulations do not compromise nasal barrier integrity (Figure 7A).

**Figure 7:**
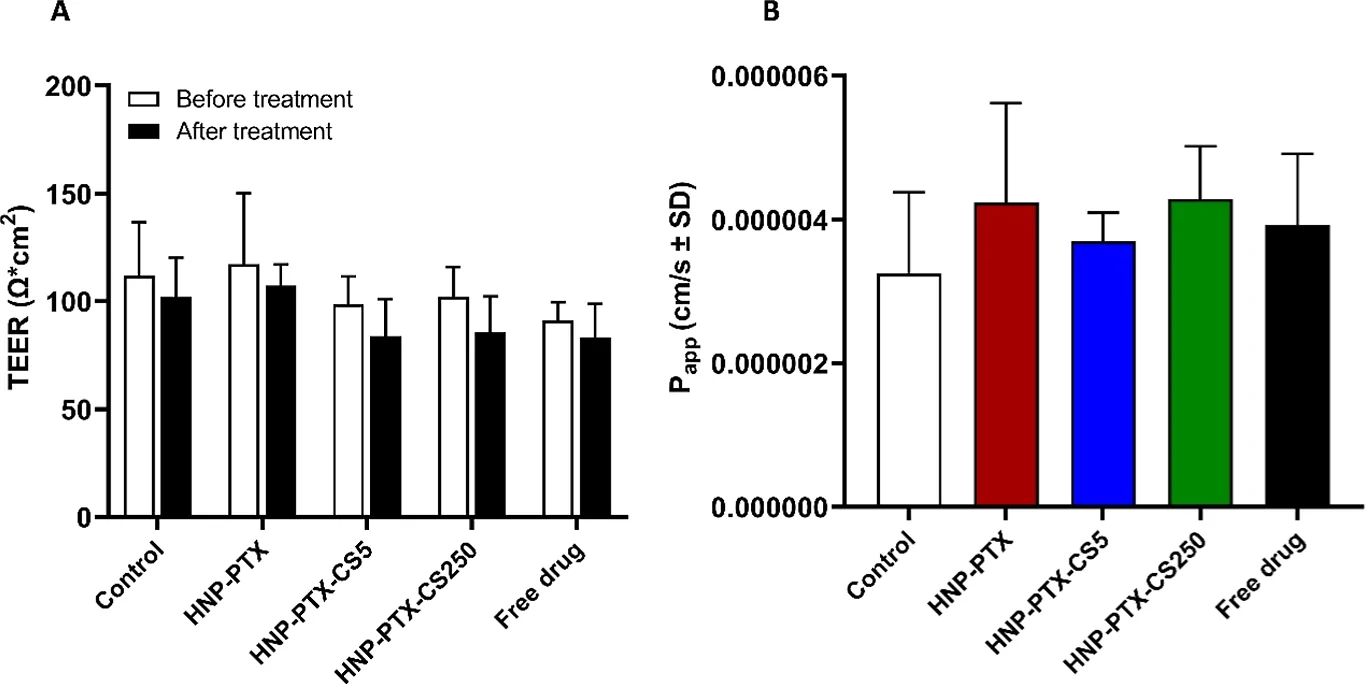
Assessing the effect of HNP–PTX formulations on RPMI-2650 nasal epithelial cells (A) TEER values were measured before and after a 4 h exposure to the HNP–PTX formulations, a control, and free drug. (B) Apparent permeability of Flu-Na over the same 4 h period. Results are expressed as the average of four replicates (*n* = 4) ± standard deviation.

In addition, fluorescein sodium was employed as a paracellular marker to assess tight junction integrity. In accordance with the TEER findings, Flu-Na permeability showed no significant differences between control and treated cells (*p* > 0.05), indicating that the HNP–PTX formulations do not disrupt the tight junctions, which are essential for maintaining the barrier’s structural and functional integrity (Figure 7B).

Overall, these results confirm that, at the tested dose, HNP–PTX formulations preserve the barrier properties of RPMI-2650 nasal epithelial cells. This finding supports the development of a safe and effective intranasal PTX delivery system and the feasibility of nose-to-brain drug delivery in neuro-oncology. However, chronic exposure studies extending beyond the 4 h period would be required before any translational claims can be made.

### 4.7 Drug Deposition and Transport Assessment Using the Air–Liquid Interface Model

Given the short residence time of most drugs in the nasal cavity due to rapid mucociliary clearance, accelerated permeation across the nasal epithelium represents a key advantage of intranasal formulations. This is particularly relevant for nose-to-brain delivery, which enhances drug availability in the central nervous system.^45^ To evaluate this effect, PTX transport from the different formulations was assessed across nasal epithelial cells cultured under ALI conditions and compared with a PTX solution as the control. The cumulative amount of PTX permeated within 240 min is shown in Figure 8A. In this study, faster PTX transport for HNP–PTX-CS250 relative to HNP–PTX was observed only at 30 min (*p* = 0.0334). Figure 8B presents the end-point distribution of PTX, including the fractions transported across the epithelium, retained on the apical surface, and internalized by the cells. A significantly higher PTX transport was observed for all HNP–PTX formulations compared with the control (PTX solution). In all groups, the apical PTX fraction was significantly lower than the intracellular fraction, except for free drug. Notably, a greater intracellular accumulation of PTX in cells treated with the free drug, which indicates enhanced internalization. Thus, the delivery system facilitates the efficient passage of the drug across cellular barriers, rather than just retaining it inside the cells. No significant difference was observed among HNP–PTX formulations.

**Figure 8:**
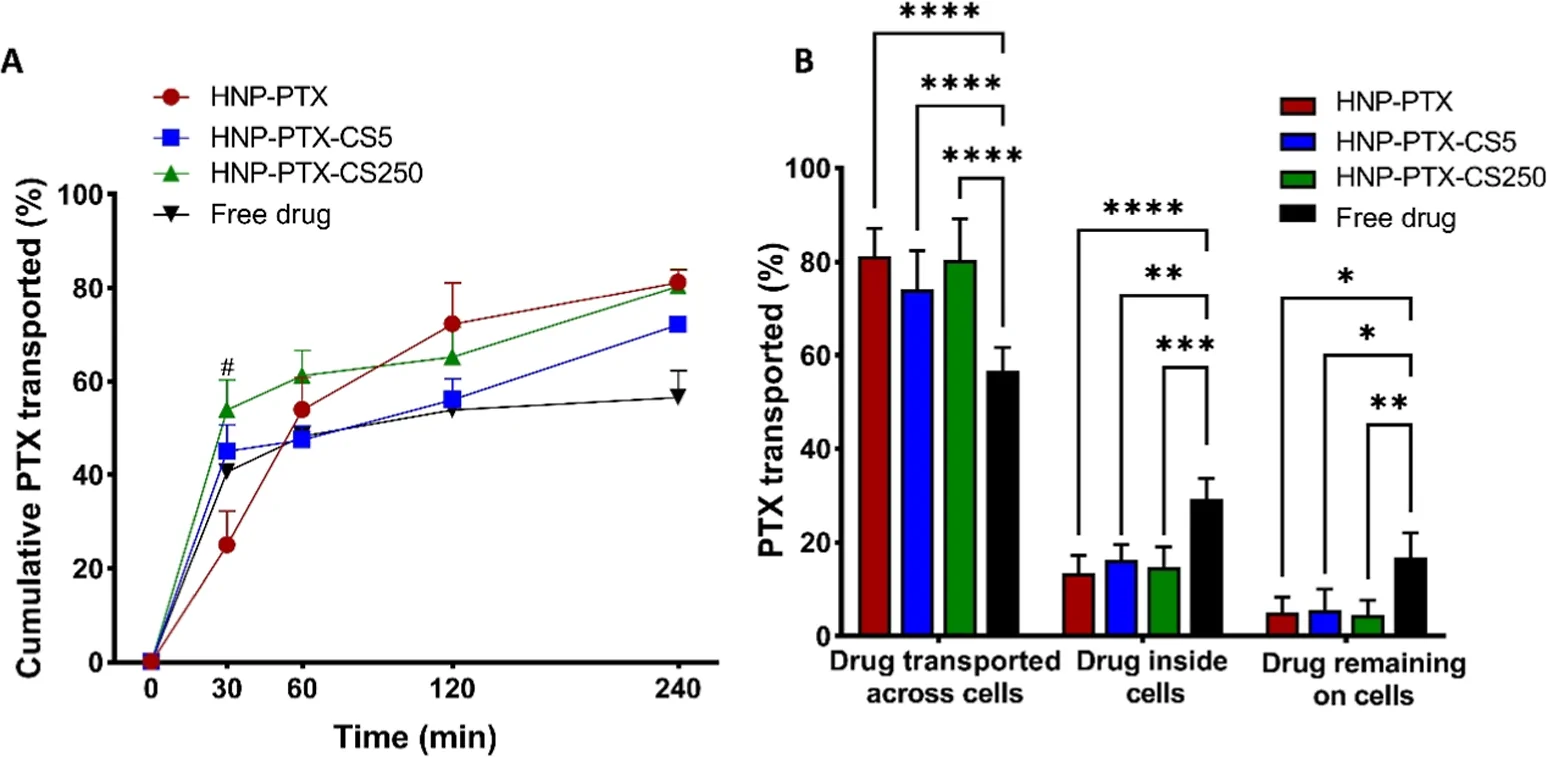
Transport profile of PTX in RPMI-2650 nasal cells after 4 h of exposure to different formulations. (A) Percentage of PTX transported from the apical layer of RPMI-2650 cells to the basolateral side over time. (B) Fractions of PTX localized in the basolateral chamber, accumulated intracellularly, and persisted on the apical side. Data are mean ± standard deviation (*n* = 3). *, **, and **** represent significant differences compared to free drug at *p* < 0.05, *p* < 0.01, and *p* < 0.0001, respectively. # represents a significant difference between HNP–PTX CS250 and HNP–PTX (*p* = 0.0334).

Although CS is known to act as a mucoadhesive polymer and permeation enhancer, no significant differences in overall transport rates were detected among the HNP–PTX formulations. Thus, under these experimental conditions, adding CS did not substantially improve drug transport across the nasal epithelium. These data further suggest that the primary driver of increased transport is the nanoparticle platform itself, overriding any additional effect of the CS coating, since the presence of polysorbate 80 and PEG chain can provide a relevant mucus-penetrating effect as previously reported.^46,47^

### 4.8 Cytotoxicity Study

The cytotoxicity of uncoated HNP–PTX was evaluated in U87 glioblastoma cells. A dose-dependent reduction in viability was observed for U87 glioblastoma cells after uncoated HNP–PTX treatment, declining from 84.5 ± 7.7% at 0.096 nM to 25.2 ± 2.0% at 7.5 μM with an IC_50_ of 0.27 nM (Figure 9A). The HNP–PTX-CS5 formulation exhibited comparable cytotoxicity against U87 cells, with an IC_50_ of 0.39 nM (Figure 9B), whereas HNP–PTX-CS250 showed lower cytotoxicity, with an IC_50_ of 12.77 nM (Figure 9C).

**Figure 9:**
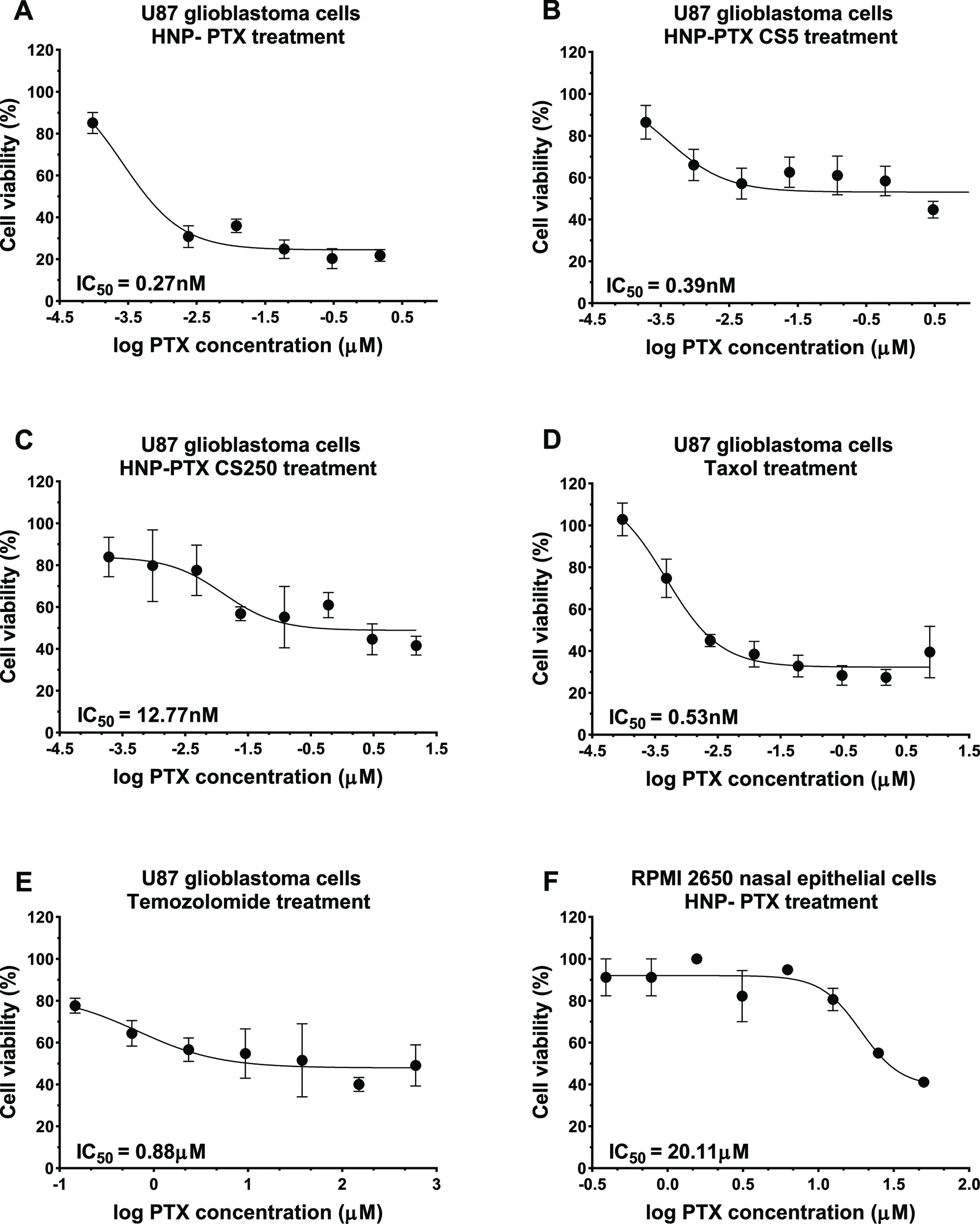
Dose–response curves and IC_50_ values for HNP–PTX, Taxol, and TMZ against RPMI-2650 nasal cells and U87 glioblastoma cells. Curves show the relationship between drug concentration and cell viability after 48 h of treatment, fitted using nonlinear regression (A) U87 treated with HNP–PTX; (B) U87 treated with HNP–PTX CS5; (C) U87 treated with HNP–PTX CS250; (D) U87 treated with taxol (free PTX); (E) U87 treated with temozolomide; (F) RPMI-2650 treated with HNP–PTX.

HNP–PTX was further compared with Taxol (free PTX) and Temozolomide in U87 cells. As shown in Figure 9D,E, the IC_50_ of HPN-PTX was approximately 2-fold lower than that of taxol, suggesting that nanoparticle-mediated delivery enhances PTX anticancer activity. Similar observations have been reported for PTX-loaded PLGA nanoparticles, which exhibit greater antiproliferative activity than taxol in U87 cells, likely due to improved cellular uptake and controlled release.^25^ Additionally, research by Hadinoto, Sundaresan, and Cheow^48^ suggests that encapsulating drugs in hybrid nanoparticles can facilitate cellular internalization via endocytosis, reduce efflux pump-mediated drug elimination, and increase intracellular drug retention, thereby enhancing cytotoxicity.

Notably, HNP–PTX also exhibited significantly lower IC_50_ values than TMZ (Figure 9A,E), indicating superior antiglioma activity compared to the clinical standard. These findings are consistent with Cancer Cell Line Encyclopedia data showing robust in vitro antiglioma effects of PTX, with an IC_50_ approximately 1400-fold lower than that of TMZ.^1,2^ Together, these findings reinforce PTX’s potential in glioblastoma therapy, especially when delivered via nanoparticle-based systems. Further mechanistic studies are required to determine whether this is due to differential uptake, metabolism, or intrinsic PTX sensitivity.

In RPMI 2650 nasal epithelial cells, uncoated HNP–PTX induced a dose-dependent reduction in cell viability, decreasing from 99.0 ± 2.5% at 0.39 μM to 41.1 ± 0.9% at 100 μM, with an IC_50_ of 20.12 μM (Figure 9F). Unloaded HNP showed no significant cytotoxicity (data not shown), confirming that the effect is attributable to PTX.

These findings demonstrate the greater sensitivity of U87 cells to HPN-PTX and indicate selective cytotoxicity toward glioblastoma cells while maintaining tolerability in nasal epithelial cells, supporting its potential for safe intranasal delivery. Moreover, the CS coating did not enhance the in vitro antitumor efficacy of PTX under the conditions evaluated. While the lower CS concentration (HNP–PTX-CS5) preserved cytotoxicity comparable to the uncoated formulation, the higher CS concentration (HNP–PTX-CS250) reduced cytotoxic potency.

## 5 Conclusions

In the present study, paclitaxel-loaded PLGA/DSPE–PEG hybrid nanoparticles (HNP–PTX) optimized via a 2^2^ factorial design produced monodisperse, core–shell particles <200 nm with confirmed thermal stability. All formulations generated nasal-appropriate sprays (Dv50 < 150 μm) and deposited in a nasal cast without throat drip. In addition, HNP–PTX increased PTX transport versus free drug without affecting TEER or Flu-Na permeability in ALI-cultured RPMI-2650 epithelial cells. In U87 cells, HNP–PTX was more cytotoxic than Taxol and Temozolomide while remaining tolerable to RPMI-2650 cells. Although HNP–PTX-CS250 showed enhanced olfactory deposition, it exhibited lower *in vitro* antitumor activity than the uncoated formulation. Overall, the uncoated HNP–PTX formulation provided the most favorable balance between physicochemical properties, nasal deposition, epithelial transport, and biological activity. These findings support its potential as a promising candidate for intranasal paclitaxel delivery and justify further preclinical *in vivo* studies to evaluate its efficacy and safety in glioblastoma models.
